# Perceived Acceptability and Experiences of a Digital Psychoeducation and Peer Support Intervention (COPe-support): Interview Study With Carers Supporting Individuals With Psychosis

**DOI:** 10.2196/27781

**Published:** 2022-02-02

**Authors:** Rachel Batchelor, Sarah Gulshan, Halpana Shritharan, Elen Williams, Claire Henderson, Steve Gillard, Luke A Woodham, Victoria Cornelius, Jack Elkes, Jacqueline Sin

**Affiliations:** 1 Population Health Research Institute St George's, University of London London United Kingdom; 2 School of Psychology & Clinical Language Sciences University of Reading Reading United Kingdom; 3 Locum GP London United Kingdom; 4 Institute of Psychiatry, Psychology & Neuroscience King's College London London United Kingdom; 5 School of Health Sciences City, University of London London United Kingdom; 6 Centre for Technology in Education St George's, University of London London United Kingdom; 7 Imperial Clinical Trials Unit, School of Public Health Imperial College London London United Kingdom

**Keywords:** eHealth, family carers, qualitative research, psychosis, peer support, web-based psychoeducation, mobile phone

## Abstract

**Background:**

Web-based mental health interventions offer a novel, accessible, and self-paced approach to care delivery to family carers (ie, relatives and close friends who support a loved one with psychosis). We coproduced COPe-support (Carers fOr People with Psychosis e-support), a psychoeducational intervention delivered via an enriched web-based learning environment with network support from professionals and peers. In addition to the rigorous investigation of the effectiveness of COPe-support on the well-being of carers and mental health outcomes, it is imperative to understand the experiences of using the web-based intervention by carers and its associated web-based implementation and facilitation strategies.

**Objective:**

This study aims to explore the experiences of carers and perceived acceptability of COPe-support and its different components, how carers found engagement with COPe-support affected their own well-being and caregiving, and the ideas of carers for improving COPe-support and its delivery to inform any future wider implementation.

**Methods:**

We conducted a qualitative study, individually interviewing 35 carers, following their use of COPe-support for 8 months through a web-based, randomized controlled trial across England. A semistructured guide with open-ended questions was used to explore the experiences of carers and perceived acceptability of the intervention and their ideas to improve the provision. All interviews were conducted remotely through mobile phones or internet communication media, audio recorded and transcribed verbatim. We used a thematic analysis framework to analyze the data.

**Results:**

Three key themes were identified: remote, flexible, and personalized support; impacts on well-being and outlook on caregiving; and future implementation and integration with existing services. Overall, carers found COPe-support a flexible source of knowledge and support from professionals and peers that they could personalize to suit their own needs and convenience. Participants described gaining self-confidence, hope, and a sense of connectivity with others in a similar situation, which helped ameliorate isolation and perceived stigma. Most importantly, COPe-support promoted self-care among the carers themselves. Participants’ experiences, use, and activity on COPe-support varied greatly and differed among carers of various ages and levels of computer literacy.

**Conclusions:**

Nearly all participants had a positive experience with COPe-support and supported its wider implementation as a beneficial adjunctive support resource for carers in the future. Any future scale-up of such an intervention needs to consider feedback from carers and suggestions for further improvement. These included having more graphics and audiovisual content materials, improving the navigation, and building in more interactional and customization options to suit various user styles, such as emoji reactions, live web-based chat, opting in and out of updates, and choosing the frequency of reminders. To ensure successful implementation, we should also consider factors pertinent to reaching more carers and integrating the web-based resources with other conventional services.

**Trial Registration:**

International Standard Randomized Controlled Trial Number (ISRCTN) 89563420; https://www.isrctn.com/ISRCTN89563420

**International Registered Report Identifier (IRRID):**

RR2-10.1186/s12888-020-02528-w

## Introduction

### Background

Family members or close friends supporting a loved one affected by psychosis (ie, family or informal carers) play a crucial role in promoting better prognosis and well-being of individuals [1-3]. However, the demands and responsibility of caregiving can make carers vulnerable to physical and mental ill-health [4,5]. Carers need access to psychosocial treatment for knowledge and support to care for their loved ones and to sustain their own well-being [6]. In recent years, with the increasing popularity of digital health interventions targeting a wide range of common mental health symptoms among the general population [7], a few clinical trials investigating such provision for carers of people with psychosis have emerged [8-11]. These web-based interventions tend to be complex multicomponent encompassing psychoeducation (ie, information focused on the health condition and its management) and web-based forums where carers can share emotional support with peers in a *closed* group, for example, COPe-support (Carers fOr People with Psychosis e-support) [12] and Relatives Education And Coping Toolkit [13]. Indeed, psychoeducation on psychosis and related care giving and problem-solving strategies, especially when integrated with peer support among carers, have been identified in systematic reviews as the most desirable ingredients for carer-focused interventions, delivered via the internet or in person [1,14,15]. In previous trials of web-based interventions targeting carers of people with psychosis, psychoeducation was the most common therapeutic approach used. The web-based medium enriched information environment allows carers to self-pick information and advice to suit their own needs and go through them at their own pace [8,11,12]. Psychoeducation and peer support can also target difficulties commonly reported among carers, including isolation, stigma, and uncertainty [16].

Web-based interventions allow for flexible access by carers, minimizing accessibility barriers, such as geographic constraints from needing to be in a particular location and time constraints from juggling multiple roles and responsibilities [17,18]. The web-based medium of delivery also facilitates autonomous use of an individually tailored package of support (ie, carers can choose how and when to use the content at their own convenience) [14,19]. Paradoxically, web-based interventions typically report much lower adherence and completion rates compared with face-to-face interventions, limiting the evidence about their effects [7,14]. Internet support groups and web-based peer forums are often highlighted as desirable features of web-based interventions for promoting social connections and mutual support in mental illness [7,20]. However, their effects, on their own or as part of a complex multimodal intervention, are inconclusive [21-23]. Although users have often identified a peer forum as an engaging element of web-based health interventions [24,25], user characteristics and their use of such forums vary widely [21,26]. Recently, Geramita et al [20] explored the applicability of the 1% rule in a computerized cognitive behavioral therapy platform, which included a patient support group. The 1% rule originated from the web-based marketing literature, suggesting that 1% of participants in web-based communities generate approximately 90% of new content [27]. A computerized cognitive behavioral therapy study [20], among other web-based health intervention trials [24,26], identified that a small number of users (approximately 10%) post most of the content in peer forums, and the remainder mainly observe activity. When considering the use of web-based health tools and services by an individual and subsequent health behavior uptake in general, Powell and Deetjen [26] proposed a new typology. In their study, they identified 6 types of web-based health users (learners, pragmatists, skeptics, worriers, delegators, and adigitals), prompting consideration of the motivation of individuals and orientations behind health-related internet use [26]. Limited evidence to date suggests that high engagement levels with peer forums or discrete elements of complex web-based interventions (eg, information and forums) are associated with better health outcomes or subjective satisfaction or acceptability [7,14,20,28-30]. At the same time, these issues highlight the challenge of implementing complex web-based health interventions that include a peer support forum element with diverse participant profiles and experiences.

Although web-based interventions present a promising opportunity to address a long-standing lack of treatment and support for carers of individuals with psychosis, they can only affect meaningful changes in their users by optimizing their engagement and facilitation strategies to ensure they get the intended benefits. Considering other challenges inherent in developing and evaluating web-based interventions (eg, safety, personalization, trust, reach, and uptake) [29], it is imperative to embed qualitative process evaluation within web-based intervention trials. While randomized controlled trials (RCTs) are the gold standard study design to establish the clinical effectiveness of an intervention, process evaluation to evaluate the experience of participants and perceived acceptability of the intervention and associated facilitation strategies can identify essential contextual factors in outcomes. For web-based interventions, the contextual factors in question are multiplied, as these interventions are designed to be used autonomously by users in their own homes. Hence, the Medical Research Council complex intervention framework advocates that a thorough process evaluation is needed to understand both the intervention and its implementation process, as experienced by the participants, and to clarify variations in outcomes under contextual influences [31].

### Objectives

This qualitative study explores carers’ experiences and perceived acceptability of COPe-support and its different components as part of the process evaluation of the COPe-support trial [9,32]. We aim to understand from the carers if and how using COPe-support affected their own well-being and the way they provided care for their loved one. With their experience of using COPe-support, the ideas of carers for improving COPe-support and its delivery were also invited to inform any future wider implementation.

## Methods

### Research Design and Setting

This study used in-depth individual interviews conducted between February 2019 and October 2020, with participants who had been randomly allocated to use the intervention, after the final follow-up data collection (ie, 8 months after the allocation), as described in the trial protocol [9].

For the RCT of COPe-support, a total of 407 family members or close friends who provided at least weekly support for a loved one affected by psychosis across England were recruited [32]. Over the duration of 2 years (ie, March 2018 to February 2020), 6 cohorts each starting 4 months apart and lasting 8 months were scheduled; when participants consented to participate in the trial, they were allocated to the next cohort scheduled to start [9]. This approach allowed us to group an optimal number of participants (ie, 40-120 participants) established from our earlier systematic reviews into each cohort, which was closed [1,14]. We believe these strategies facilitate peer-group building, thus enhancing the web-based elements of the intervention. Half of the participants were randomly allocated to the intervention arm, that is, access to COPe-support for 8 months, which included being able to post on the peer and expert forums for the initial 4 months (termed the active intervention use period), in addition to usual care. The remaining participants were randomized to receive a web-based noninteractive information bank as an attention-matched control, also with usual care [9].

### The Intervention

**Figure 1:**
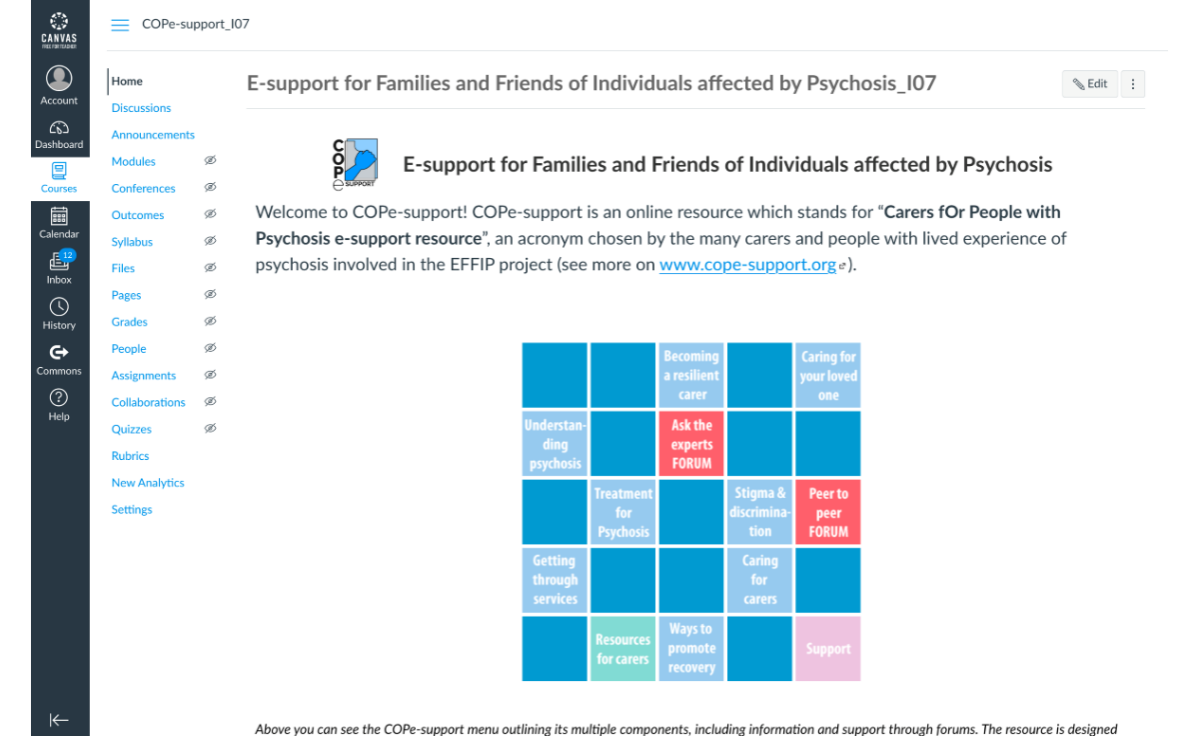
COPe-support (Carers fOr People with Psychosis e-support) home page.

**Figure 2:**
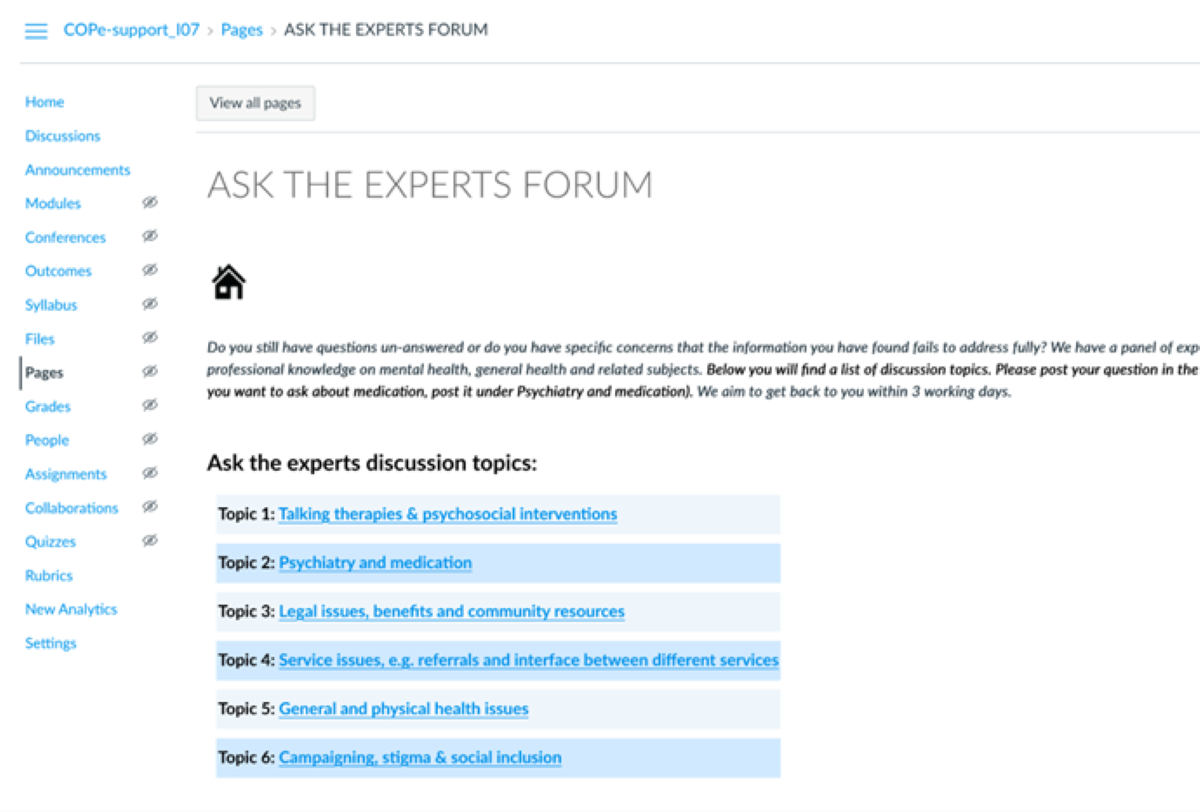
Ask the Experts forum webpage.

**Figure 3:**
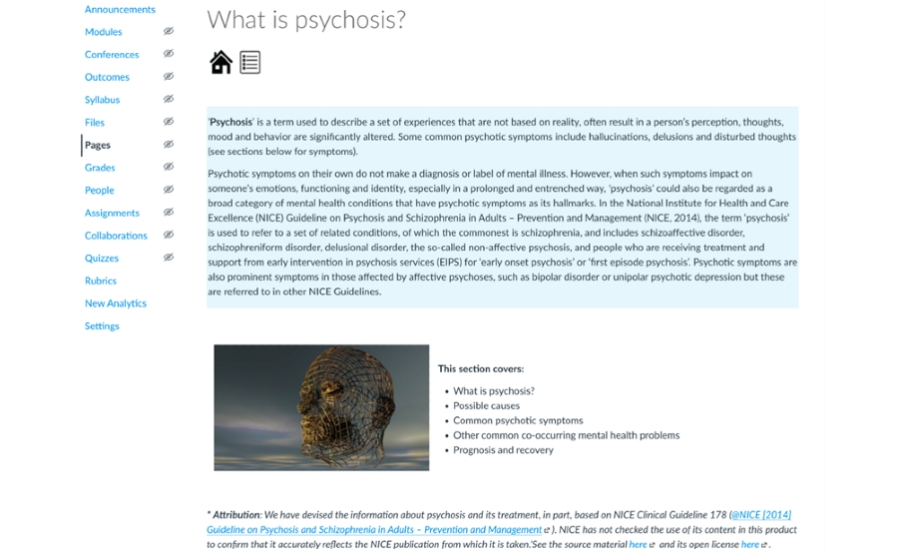
Information on psychosis on COPe-support (Carers fOr People with Psychosis e-support).

The web-based intervention COPe-support was coproduced using participatory research methodology as described elsewhere [12]. COPe-support was delivered through a web-based, enriched environment platform that carers could access through a web browser using a computer or a laptop or through an app on smartphones or tablets [9,12]. COPe-support comprises multiple components, including psychoeducation on psychosis and related caring issues, guidance on well-being promotion information and exercises, a *Resource for carers* section signposting to a wide range of external resources weblinks; and 2 web-based forums (one called *Ask the Experts,* where participants could post questions for advice from a panel of experts and the other called *Peer to Peer* for participants to exchange views with one another; see Figures 1-3 for screenshots of COPe-support components). Throughout the study period, a web-based facilitator (an experienced mental health nurse, JS) monitored and moderated all the interactive functions of COPe-support. A weekly email update was sent through the COPe-support platform to all participants for the first 4 months of the study period, which was regarded as the active use period. For security and confidentiality considerations, participants were required to follow a set of ground rules, including using a self-chosen pseudonym and observing confidentiality principles by not sharing any identifying information about themselves and their cared-for person on the COPe-support platform. The web-based intervention platform had an inbuilt use data recording system for log-ins, time spent, and the number of posts made by each participant.

### Participants

The inclusion criteria for the RCT specified family members, relatives, and close friends who were aged ≥18 years, had at least weekly contact with the cared-for person (in any form ranging from face-to-face to social medical communications), living in England, able to communicate in English in usual web-based communications, and had daily access to the internet, including emails [9]. The inclusion criteria for this qualitative study specified that participants had (1) been randomized to the intervention arm and (2) completed the final RCT follow-up (8 months). Furthermore, purposive sampling was used to identify about 20% of participants in each cohort intervention group from across the *2*-year study period to ensure representation of those with different demographic factors and different levels of use of COPe-support. As previous literature shows that female and White individuals tend to form most of the participants in intervention trials targeting carers for a loved one with psychosis in Western countries [1,3,6,14], we prioritized male carers and those from ethnic minority backgrounds in approaching potential participants. To examine use, we followed the approach by Valentine et al [33] in categorizing participants into levels of use based on the overall number of log-ins by participants to the COPe-support platform over the 4-month active intervention use period. We categorized participants into three use groups as follows: (1) noncompliers, participants who had not activated their log-in or only logged in once; (2) moderate users, those who had logged in between ≥2 and ≤10 times; and (3) high users, those who had logged in >10 times. Within the use categories, we also considered whether participants had forum posts. Thus, participants in the three use groups were further categorized into the following: (1) passive users, participants who did not post, and (2) active users, participants who made at least one forum post, in accordance with previous web-based forum research [34,35]. Participants representing the various demographic considerations and use levels were then contacted via email and invited to participate in an individual interview. A total of 43 participants were invited; 81% (n=35) of the participants agreed, whereas 19% (n=8) of the participants did not respond to the invitation with *2* reminders (their reasons for not responding were not provided).

A total of 35 participants were interviewed and included in the study. The mean age of the participants was 56 years (SD 13 years, range 23-73 years). Most of the participants interviewed were White (29/35, 82%, White British and 2/35, 6%, other White), whereas 6% (2/35) of the participants each described themselves as Asian and Black. Approximately 63% (22/35) of the participants were women, and most (23/35, 66%) cared for a male person. Parents comprised most of the participants (23/35, 66%), followed by partners (7/35, 20%), whereas siblings (2/35, 6%) or close friends (3/35, 8%) formed the remainder. According to the data on their caregiving roles and activities provided by the participants, the mean age of the cared-for persons was 35 years (SD 14 years, range 17-66 years). Just over half (18/35, 51%) of the participants reported that their cared-for persons first became unwell with psychosis <5 years ago, whereas 11% (4/35) of the participants described their loved ones had their first psychotic onset over 20 years previously, and the remainder (13/35, 37%) had been caring for between 5 and just under 20 years. Approximately half (17/35, 49%) of the participants lived with their cared-for person, and 40% (14/35) of carers reported spending over 20 hours per week in caregiving activities. Table 1 provides a summary of the participants’ demographic, caregiving, and use data.

**Table 1:** Summary of participant use and demographic information categorized by use groups.

Users and pseudonym				Cohort (start time)		Sex of carers		Age of carers (years)		Relationship with CfP^a^		Sex of CfP		Age (years) of CfP		Overall weekly log-ins^b^		Overall page-views^c^		Posts made
**Noncompliers (who have not logged in or only logged in once throughout the 4 months)**																				
	**Passive users (<1 post made)**																			
		Mark	October 18		Male		53		Parent		Male		27		1		3		0	
		Alexandra_1	February 19		Female		62		Parent		Male		31		1		8		0	
		Ahmed	February 19		Male		41		Partner		Female		40		1		3		0	
		Aaron	June 19		Male		23		Partner		Male		22		1		40		0	
		Sally	October 19		Female		72		Parent		Male		36		—^d^		0		0	
		Anna	October 19		Female		50		Parent		Female		20		—		0		0	
**Moderate users (who have logged in between ≥2 and ≤10 times in different weeks)**																				
	**Passive users (<1 post made)**																			
		Fern	June 18		Female		57		Sibling		Male		65		4		163		0	
		Martin	June 18		Male		63		Parent		Female		27		4		83		0	
		Summer_2	October 18		Female		54		Parent		Male		17		6		238		0	
		Faye	February 19		Female		68		Parent		Male		38		4		561		0	
		Alfred	February 19		Male		55		Partner		Female		43		5		263		0	
		Sam	June 19		Female		71		Parent		Male		42		4		137		0	
		Polly	February 20		Female		70		Parent		Male		41		8		677		0	
		Hamish	February 20		Male		55		Parent		Male		30		2		20		0	
		John	February 20		Male		50		Partner		Female		59		6		482		0	
		Edward	February 20		Male		33		Partner		Female		30		2		195		0	
	**Active users (≥1** **post made)**																			
		Katrina	February 19		Female		62		Sibling		Male		57		2		533		10	
		Alexandra_2	June 19		Female		72		Parent		Male		32		6		930		15	
		Alexandra_3	June 19		Female		58		Parent		Male		20		4		602		22	
		Ben_2	June 19		Male		69		Parent		Male		35		10		974		11	
		Felix	October 19		Male		42		Stepparent		Male		17		3		226		3	
		Abbie	October 19		Female		54		Parent		Female		27		5		212		4	
		Molly	February 20		Female		50		Parent		Male		17		7		248		4	
		Sophie	February 20		Female		27		Friend		Female		26		3		63		1	
		Louise	February 20		Female		73		Parent		Male		40		6		206		2	
**High users (those who have logged in >10 times in different weeks)**																				
	**Active users (≥1 post made)**																			
		Matthew	February 19		Male		46		Partner		Female		44		15		1125		1	
		Flossie	June 18		Female		58		Parent		Male		28		13		654		3	
		Tony	June 18		Male		43		Partner		Female		41		19		2602		31	
		Summer_1	October 18		Female		57		Parent		Male		25		11		311		3	
		Alex	October 18		Female		56		Parent		Male		24		15		554		3	
		Ben_1	February 19		Male		66		Parent		Male		37		13		715		7	
		Eleanor	February 19		Female		63		Friend		Female		63		13		967		29	
		Abby	October 19		Female		67		Partner		Male		66		12		354		4	
		Maryam	October 19		Female		67		Parent		Female		26		11		1154		10	
		Imogen	February 20		Female		62		Parent		Female		30		14		1227		7	

^a^CfP: cared-for person.

^b^Number of weeks with log-ins across the 4-month active intervention use period.

^c^Total page-views across the 4-month active intervention use period.

^d^Has not activated the log-in.

Multiple participants from each of the different use groups across cohorts were interviewed. All participants were interviewed shortly after their access to the intervention platform ceased (ie, at the 8-month follow-up), although their last access to the platform varied widely. Of the 35 participants, 17% (n=6) participants were classified as noncompliers. All noncompliers were passive users (passive noncompliers). Many participants (19/35, 54%) were classified as moderate users, 53% (10/19) of whom were passive (passive-moderate users) and 47% (9/19) were active (active-moderate users). High users comprised the remaining 28% (10/35) of participants, all of whom were active within the COPe-support forums (active-high users).

### Data Collection

All interviews were conducted remotely, suiting the preferences of participants for either phone or internet-facilitated interviews (using Skype [Microsoft] or Microsoft Teams). No face-to-face interviews were used, as all participants had joined the web-based trial of a web-based intervention, with no requirement for in-person contact. Author JS conducted all interviews. Informed written consent was obtained from each participant through the web-based study platform before the interview. At the beginning of each interview, we asked the participants to confirm their consent orally, including for the interview to be audio recorded. All interviews were audio recorded, apart from 3% (1/35) of the participants who opted for their interview recorded by written notes instead.

The interviews followed a topic guide that was devised by the Project Reference Group members, including individuals with lived experiences of psychosis or caring for a loved one with psychosis, who had been involved in developing the intervention [12]. In line with the objectives of this interview study, the interviewer asked open-ended questions to explore the experiences of participants and their views of COPe-support, any specific features of the intervention that they liked or disliked, and the barriers to and facilitators of their access and use of COPe-support, including the facilitation strategies used. The interviewer asked the participants to reflect on their subjective evaluation of the impact of using COPe-support on both themselves and their caregiving experiences. Finally, the interviewer asked the participants for their views and ideas for a plausible wider implementation of COPe-support in the future. The topic guide, which includes the semistructured interview questions and prompts, is presented in Multimedia Appendix 1. Interview times ranged from 14 to 49 minutes and a total of 1117 minutes of data were transcribed.

### Data Analysis

The audio recordings were transcribed verbatim. Only transcribed anonymized textual materials were used for the analysis. The data were analyzed in 4 phases using thematic framework analysis [36], with the software NVivo 12 (QSR International) [37]. In accordance with the thematic framework analysis, we commenced the data analysis once the first qualitative interview had been completed and transcribed. To ensure the analysis was grounded in the data and the exploration of the experiences of participants was driven by the emerging results, the interviews and analysis were performed in parallel so that the identified themes and framework of analysis could be tested and validated in latter data.

In the first analysis phase, the authors (JS, S Gulshan, HS, and RB) familiarized themselves with the data by rereading the transcripts and noting interesting aspects. In the second phase, 2 authors (S Gulshan and HS) coded all the data, and a third author (RB) coded 20% of the data independently. The data coded by the third author was selected based on user type and demographics, to ensure that all groups across the full sample were represented. Open (unrestricted) descriptive codes summarizing text segments were applied across the data set. The codes were discussed and reviewed by the authors through several iterations. In the third phase, initial themes and subthemes reflecting broad units of common ideas were formed by grouping relevant codes. These were compared by reviewing the entire data set as well as within individual cases. In the fourth and final phase, the authors (RB, S Gulshan, HS, EW, and JS, all women) cross-referenced, discussed, and clearly defined the themes and subthemes and their interrelated links over several meetings. We used a combined inductive and deductive approach to coding and selecting themes throughout the analysis process [38]. Initially, we used inductive coding, driven by the data (ie, the experience of participants or the way they assigned meaning to their perception of using COPe-support). Nonetheless, as the study aimed to explore the participants’ perception of specific elements, functions, and facilitation strategies related to the web-based intervention, we also coded the data deductively with reference to previously reported findings as reported in the literature on wider web-based health interventions and those targeting carers for individuals with a mental illness. These concerned web-based content, forums, facilitation, and perceived safety and security and were explored by questions within our interview topic guide [8,14,24]. Suggested improvements specific to COPe-support were coded deductively using the ideas generated from the views expressed by the participants. Iterative analysis of the transcript showed that saturation of data was achieved as the final 2 interview transcripts produced no new themes or subthemes [39].

### Ethics Approval and Consent to Participate

This study, as part of the overall RCT, was reviewed and approved by the South Central—Oxford C Research Ethics Committee (reference: 18/SC/0104) and the Health Research Authority (reference: IRAS 240005). Before study participation, all participants were required to view and give consent on the web to the information provided in the participant information sheet.

## Results

### Overview

**Figure 4:**
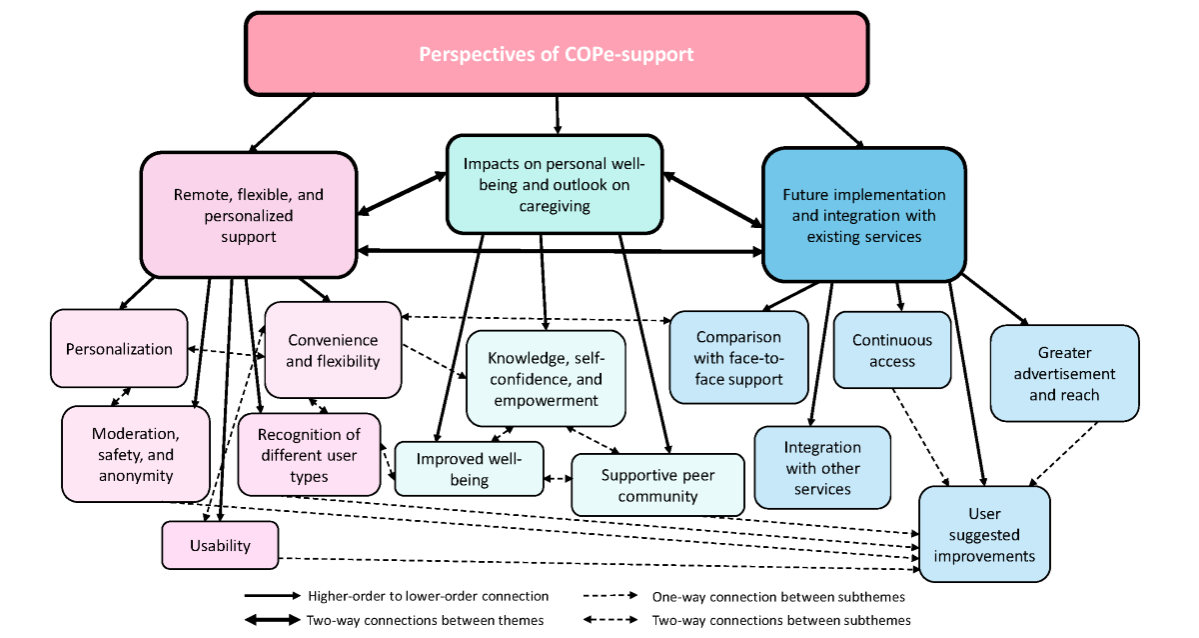
Coding tree summarizing the interrelated themes and subthemes. COPe-support: Carers fOr People with Psychosis e-support.

In total, 3 main themes were identified, with each theme divided into subthemes to comprehensively capture the phenomenon explored. The three main themes were as follows: (1) remote, flexible, and personalized support; (2) impacts on well-being and outlook on caregiving; and (3) future implementation and integration with existing services (Figure 4). A brief summary of each theme and subtheme is provided in Multimedia Appendix 2.

### Remote, Flexible, and Personalized Support

This theme covered the experiences and perspectives of carers using COPe-support, with particular regard to navigation, safety, and usability. This theme incorporated 5 subthemes as follows.

#### Personalization

Carers mostly appreciated that they could choose and focus on specific content on COPe-support, suiting their own circumstances and needs. Several carers also valued the ability to choose their own pseudonyms. Although sharing a common caregiving role, carers recognized that they each have specific interests and needs based on their cared-for person’s presentation, treatment, and a range of caregiving factors. For instance, for some carers, information and advice on getting through the benefits system could be a priority at the time, whereas others were after a summary of research evidence of a new treatment:

Whereas the stuff from the website was quite helpful and we could tailor it to our own sort of thing. Yes, it was just nice.

Aaron; male, partner, passive noncomplier

Some carers discussed a preference for having a greater sense of independence and choice regarding communication in the forums. This included being able to opt in and out of updates and chose the frequency of reminders.

#### Convenience and Flexibility

Many carers appreciated the convenience of having the information and resources they needed in one place and being able to revisit information and access information at any time and place in day-to-day life. Carers particularly valued the flexibility of their use of COPe-support. This included having autonomy over their use and posting without having to adhere to engagement targets, as well as being able to pick out relevant information at their own pace. Several carers found revisiting and downloading the information for future reference particularly useful:

Yes, I mean anything that was easier to download and keep for reference, I mean it’s always good to have reference material.

Ben_2; male, parent, active-moderate user

Carers particularly appreciated having access to a range of professionals and found it fascinating to receive different perspectives from experts with various experiences and knowledge. Most carers also appreciated the convenience of expert knowledge on COPe-support. Several considered this novel and felt it addressed the lack of access to experts in existing services for loved ones. Carers valued the opportunity to ask specific questions at any time and received prompt and thought-out answers. A couple of carers noted that this was in contrast to their experiences of feeling rushed within appointments with professionals:

I think looking back to seeing the doctors and the psychiatrists you feel a bit rushed and they haven’t got time to think about it much but if it’s sent as a question you feel someone has taken time to give you an in-depth answer.

Abby; female, partner, active-high user

#### Moderation, Safety, and Anonymity

Being anonymous helped many carers feel more comfortable interacting on forums. Most carers felt that anonymity helped to protect the privacy of their loved one with psychosis and did not affect the community feel on COPe-support:

Anonymization probably is quite important because if you are posing questions or comments about your experience as a carer, you inevitably have to talk about that person and they might not like you doing that.

Maryam; female, parent, active-high user

Carers particularly appreciated the ground rules (eg, being respectful and not mentioning identifying information) and forum moderation (eg, checking and approving content), providing reassurance that the forums comprised a safe environment. Some carers expressed appreciation and preference for professionals (as used in the trial) over hypothetical carer moderation, providing the professional understood the needs of carers, to help increase the accuracy of information, dispel potentially misguided beliefs, and manage disagreements.

Weekly emails tended to have a positive impact: carers felt they not only served as a reminder for the intervention but also that someone cared. Some carers described themselves as looking forward to or smiling at the emails. Overall, the carers shared a sense that COPe-support was safe and trustworthy, as reflected in the following:

It’s having a trusted site to look at and knowing that if you put anything on it it’s a safe place.

Abbie; female, parent, active-moderate user

#### Usability

Mixed experiences were shared regarding the usability of the COPe-support. Some carers felt confident owing to good computer literacy or previous experience with similar platforms, whereas others described barriers, such as age and poor computer literacy. Carers appreciated accessing COPe-support on different devices (eg, computers, laptops, or mobile phones), with some finding devices with larger screens easier to navigate. Most carers described an adjustment period during which they initially struggled with navigating the interventions but adjusted and grew in confidence over time:

I did start to get a bit more used to [navigating] after a while but to begin with I did find it complicated.

Summer_1; female, parent, active-high user

#### Recognition of Different User Types

Many carers expressed awareness of different user types on COPe-support. Carers tended to distinguish between enthusiastic (active) users whose names frequently appeared within forums and other (passive) users who tended to observe. Some active users reported focusing on the peer and expert forums and felt these aspects in themselves made COPe-support *powerful* (Eleanor; female, friend, active-high user and Alexandra_3; female, parent, active-moderate user)*.* A couple of active users were unaware of being particularly active and felt unsure of how many people read their posts. Most passive users were aware that they had not posted and found reading what others had to say useful and knowing the forums were there if needed comforting in itself:

I guess there are some people who are going to be very active on there and discuss things a lot and then there are going to be people who are very quiet on there...It doesn’t mean to say that they’re not taking it all in and getting something from it...I also regret slightly now that I wasn’t a bit more active at the same time.

Summer_2; female, parent, passive-moderate user

### Impacts on Well-being and Outlook on Caregiving

Three subthemes represented the impacts on well-being and outlook on caregiving experienced from participating in COPe-support.

#### Knowledge, Self-confidence, and Empowerment

Many caregivers felt that COPe-support provided comprehensive, relevant, and helpful information across various important topics. New carers found the information especially suitable for their first time learning about psychosis. For others with existing knowledge, the information supplemented the resources they had previously accessed. Although some felt COPe-support had enhanced their knowledge and skills enough to not require further support, others appreciated the signposting to other sources and local and national services to further support their loved ones:

I’ve got 99% certain I will either get signposted in the right direction or find what I want rather than Googling and going through different websites and trying to find the same information.

Faye; female, parent, passive-moderate user

Some carers felt that the information was quite generic, outdated, and repetitive. Moreover, some newer carers initially found the amount of information overwhelming, although they reported adjusting and learning over time:

It’s also a strength is the fact that once you are in the program you realize just how comprehensive and detailed it actually is and that this could be a bit daunting initially for people signing up.

Eleanor; female, friend, active-high user

Most carers felt that the tone of the experts was just right: not pressurizing or patronizing, yet empathetic, respectful, and comforting. Understandable language (eg, no acronyms, abbreviations, and technical terms) was also used to explain complex information in an understandable way. However, several carers felt that the answers were sometimes generic or vague, although they appreciated that the experts were not aware of the full situation of their loved ones and still found the suggestions helpful. The information provided and knowledge gained subsequently empowered them to seek further conversations with mental health professionals caring for loved ones, as expressed by a participant:

They’ve not been able to provide really specific answers sometimes because obviously they don’t know our situation but the fact of it is they’ve been able to signpost or suggest something that you maybe hadn’t thought of.

Alexandra_3; female, parent, active-moderate user

Some carers discussed the lack of preparation for caring roles and the ongoing self-doubt surrounding doing the right thing or supporting their loved one in a helpful way. These carers felt by gaining information, resources, and knowledge on COPe-support had better equipped them and also improved their self-confidence in their ability as carers:

I’m sure it’s given me more confidence as a carer because I’ve got more information and that also becomes a part of how I care for my daughter and talk to the family and others as well.

Maryam; female, parent, active-high user

#### Supportive Peer Community

One significant benefit identified was a sense of belonging to a supportive peer community, without ever seeing or knowing one another. Many carers discussed feelings of loneliness and isolation experienced by them. Reading the resources and forums showed carers that others were experiencing similar and relatable difficulties, helping them feel less alienated, isolated, and detached:

Sometimes when you are a carer you think you are alone. When you go to these groups or you do these things, you realise you are not. It makes a difference.

Anna; female, parent, passive noncomplier

Carers also reported feeling more connected and having a sense of solidarity and unity with others to proceed on the caring journey. Carers valued having a sense of community, group alliance, and connection, which naturally arose from sharing similar experiences and challenges and feeling mutually understood, something they often lacked in their own lives. This was made explicit by the following:

Being able to see that people are getting some support and that it normalizes the issues that we don’t talk about.

Abbie; female, parent, active-moderate user

Most carers valued being linked with other carers, especially new carers who felt shell-shocked and craved speaking to others in a nonjudgmental environment. Many carers appreciated the opportunity to learn from peers, including practical tips, advice, and awareness of differing carer experiences. Some also valued the opportunity to help other carers and the positive feelings that came with that:

But in the main I found the whole thing quite helpful especially for the first month or so when I could see or read about everyone else’s problems and some were similar to mine and some of the advice they gave if you know what I mean.

Ben_1; male, parent, active-high user

In addition to creating a community and reducing loneliness, many carers noted that reading posts from other carers also helped normalize and validate their feelings and experiences. The intervention content and forums also helped to normalize concerns, fears, and often stigmatized psychosis-related topics that carers often found difficult to talk to people in their personal lives about. Such normalization and validation subsequently helped carers feel less overwhelmed:

Yes, I think I found it really helpful as well because some of the ways that it was designed with the different subjects helped as well to make me think oh yes well this experience I’m having is normal, which is like there was, how it was set up the program it had stigma.

Eleanor; female, friend, active-high user

Some carers felt that the expert and peer support forums provided hope, particularly in instances where carers were able to provide lived accounts and reassurance of particular aspects and situations improving over time. Some carers especially valued reminders that their loved one is still their loved one and reflected that kind words provided *light*, led to a feeling of hope:

In some respects, it made me feel a bit better because other people are going through not completely the same as me but very similar as me and they’ve managed to get through it, etc.

John; male, partner, passive-moderate user

#### Improved Well-being

Carers recognized that the COPe-support was specifically designed for them. Some carers discussed how COPe-support not only provided support for the well-being of their loved ones but also their own. This included recognizing the importance of supporting their own needs, focusing on self-care, and fostering healthier routines, such as improving their diet, fitness, and sleep hygiene:

It was just really, really helpful to learn how I can manage my well-being in terms of trying to support myself in terms of trying to help the person I’m caring for...like I said it has made a really big difference to my well-being and my partner’s well-being and it has been a lifeline.

Edward; male, partner, passive moderate user

I have actually changed my eating this last few months as a direct result of the site, so that’s quite something.

Alexandra_3; female, parent, active-moderate user

Even the concept that COPe-support had been designed specifically for carers helped carers recognize their support needs were valid and acknowledged, reducing guilt associated with personal help-seeking. Some carers described how COPe-support had provided personal space and time to reflect on their personal journey as carers, get more in touch with their emotions, and listen to the reflections of others:

Even just using the questionnaires at times were good for me because it made me sit and focus a little bit on where things were at...and actually think about how I was feeling.

Alexandra_1; female, parent, passive noncomplier

### Future Implementation and Integration With Existing Services

The following subthemes reflect the perspectives of carers surrounding the future implementation of COPe-support and integration with existing services. This includes suggested improvements for COPe-support.

#### Comparison With Face-to-face Support

Compared with face-to-face support for carers, the perspectives of COPe-support were mixed. Although some expressed a preference for traditional means of delivery, others preferred web-based platforms and ideally a blended approach. Barriers to face-to-face support, including geographic factors, family life, funding and time constraints, and the benefits of web-based delivery in minimizing these barriers were discussed by some carers. Other carers considered barriers to web-based interventions, including age and a desire to personally meet carers and be able to sit with others going through similar situations. This is expressed as follows:

And I think that e-support is definitely a very, very useful, well it’s a very good use of technology for people who have computers or phones and have the confidence to access stuff. You can’t beat that one-to-one when you need it, you can’t beat that.

Faye; female, parent, passive-moderate user

#### Integration With Other Services

Several carers commented that, given the funding restrictions on existing services, implementing COPe-support could *only be a benefit*. Some carers highlighted that participating in COPe-support addressed their concerns surrounding interventions for carers and motivated use of other services, such as face-to-face groups and courses for carers. Although some felt the support they had received through COPe-support was sufficient for their needs, others emphasized that COPe-support should serve as an adjunct to existing services rather than a replacement:

It also encouraged me to join a carers and coping course...I think it’s made me question why I would find it so hard...to sit in a group with other people and hear about what’s been happening to them, so yes I’m definitely looking forward to going to a six-week course at the end of this month.

Summer_2; female, parent, passive-moderate user

#### Continuous Access

Perspectives on the length of time to access COPe-support were mixed. Some felt they had received access for just the right amount of time to remain engaged and gain optimal benefits as a carer. However, some desired a longer use time. Several carers highlighted that as caring can be a long and complex journey, it would be reassuring to be able to revisit information and know they would be able to use it and have instant access to support in the future if new challenges arise (ie, dip in and out):

People have different periods of crisis. You would not want to have the sense of support suddenly be taken away.

Martin; male, parent, passive-moderate user

To allow for continuous access to carers’ needs, some suggested being able to self-refer back into the intervention if necessary or have continual access and be able to opt out when they felt they had used it enough:

It was very good, too good; hence I asked if I can enroll again...it was a lifeline for me...COPe-support came along and gave me all the help and support I’ve ever wanted.

Summer_1; female, parent, active-moderate user

#### Greater Advertisement and Reach

Some carers reflected on coming across COPe-support *by chance* and emphasized a need for greater advertisement to reach more carers if it was to be rolled out widely in the future. Several advertising and promotion routes have been suggested, including local authorities and social services, charities, general physician surgeries, existing services for carers and trust websites, noticeboards, and newsletters. Awareness among health and social care professionals was also noted as important, with potential screening for the well-being of carers and onward signposting to COPe-support recommended. Suggestions for ways to reach carers include the following:

When you roll it out into various Trusts and it goes further that’s where it needs to be as well. There are a number of options there.

Mark; male, parent, passive noncomplier

They always ask at the GP surgery when you register or every so often they’ll say are you caring for anyone and it could be quite helpful to maybe signpost it at that point.

Aaron; male, partner, passive noncomplier

#### User Suggested Improvements

Many carers have proposed improvements for COPe-support. Some were about the way information was presented, which some found, at times, overwhelming and off-putting (ie, *too much on the screen sometimes*). To reduce confusion, fewer chunks of text and more graphics and visual aids or *see more* dropdown options were recommended:

I suppose what I’m trying to say is even a little, you need to have something...if you want to do mindfulness it needs to have a little picture, it needs to be more visually stimulating.

Molly; female, parent, active-moderate user

With regard to forum communications, although some described freely writing open posts as cathartic, several carers reflected on an emotional burden arising from posts. At times, carers found posts distressing to read and that they could generate worries. Thus, providing a general warning of content causing potential distress and content warnings for particular comments was recommended:

There were things where...you know, there were things that triggered me to think about things and thought this maybe something worth sharing.

Matthew; male, partner, active-high user

Moreover, some caregivers desired more ongoing conversations. Thus, suggestions for a chatroom or befriender element were made by a few carers to help build stronger connections. Some carers reported it was hard to relate to others given different life circumstances (eg, having several children to care for too). Hence, a couple of carers recommended brief profiles with basic, yet nonidentifying, information (eg, sex, caring responsibilities, relationship, or living situation) to provide advice and support, as well as seeking relatable content. However, when certain forum topics received a good number of posts, one common problem that arose was having to go forward and backward among pages and scrolling excessively to see forum comments. This was described by an active user as follows:

I remember the format of the message threads when you had five or six interactions or replies on the same thread it becomes almost impossible to read on the phone because you have to scroll down and the indentation starts going to the right.

Tony; male, partner, active-high user

Hence, for navigating the forums and the COPe-support content overall, *frequently viewed* and *recently viewed* buttons were recommended by some carers. Some would also like to be able to choose which posts on the forum to expand. Most found the instructions for navigating COPe-support clear, although some would have appreciated an opt-in for 1:1 guidance.

Finally, to encourage engagement, some carers noted that they would have appreciated some additional prompting after periods of inactivity. Several passive users regretted not using the forums more and reported barriers to posting, including their busyness, mental state, difficulties expressing their feelings, worries surrounding sharing with unknown people, and experiencing hesitation and self-doubt. Some carers suggested having rolling discussion topics and implementing alternative options (eg, emoji reactions) to facilitate forum engagement:

If that [thumbs up or other emojis for acknowledgement] feature had been available and I’d seen a couple of thumbs up to the things I’d posted I think that would have been great...And maybe that’s a stepping stone as well they start by just a few reactions, emoji reactions and then it’s small steps. They can do that the first time and then maybe the next time they will write a few words.

Felix; male, stepparent, active-moderate user

## Discussion

### Principal Findings

This study aimed to explore the following: (1) carers' experiences and perceived acceptability of COpe-support and its different components; (2) how they found engagement with COPe-support affected their own well-being and caregiving; and (3) ideas of carers for improving COPe-support and its delivery to inform any future wider implementation. Notably, this qualitative study is one of the first to explore the experiences of carers of individuals with psychosis by using an entirely web-based psychoeducation and peer support intervention, coproduced by carers and people with experiential expertise. Experiences of participants were predominantly positive with COPe-support, and carers identified a range of benefits from using the intervention. Nonetheless, the carers highlighted some key areas of improvement. Overall, three themes were identified, each addressing one of the objectives of the study as follows: (1) remote, flexible, and personalized support; (2) impacts on well-being and outlook on caregiving; and (3) future implementation and integration with existing services.

Overall, the subjective experiences of COPe-support among carers were positive. In addition to the web-based gains provided by COPe-support, such as improved accessibility, flexibility, and anonymity, participants also reported that the intervention was beneficial in providing access to a rich repertoire of credible information [8,11,12] and fostering personal development by enhancing their self-confidence and understanding. Our results indicate that COPe-support was perceived as a crucial resource to reinforce feelings of empowerment in carers while reducing their sense of isolation. COPe-support also prompted carers to prioritize their own well-being. These impacts motivated some carers to access further support and engage more with professionals, indicating additional long-term benefits [1,3,6,14,15].

Notably, our themes and subthemes should be recognized as a set of interconnected and interacting constructs to be considered in the overall design (eg, content) and facilitation (eg, moderation) of web-based interventions, such as COPe-support [8]. For instance, carers would only enjoy interacting on the web-based forums, provided they felt safe and supported through specific implementation strategies. Carers would be less likely to see the essential intervention contents should access and navigation be less than facilitative.

Moreover, similar to earlier studies on web-based interventions with a forum component [20-23,33], we found that the use by carers in terms of numbers of posts and log-ins does not always align with their perceived acceptability and usefulness of the intervention. Although the carers who actively initiated posts themselves were eager to see more exchanges on the forums, many others found benefits in being passive observers. Some carers identified that anonymous participation on web-based forums allowed them not to feel pressurized to participate in a certain fashion as in a face-to-face group setting. Many carers described finding resonance, connections, and solidarity from the peer and expert forums without making a post themselves, although some identified that they would have made posts if given more time or if a specific question came up.

### Future Directions

The experience of participants seemed partly dependent on factors, such as their own demographic profile (eg, length of time as a carer and age) and preferences for particular delivery formats and computer literacy as highlighted in previous studies [14,26,40,41]. It is imperative to incorporate these perspectives in considering how best to further refine COPe-support and its facilitation. Upon future implementation, several advertisement routes were recommended to increase the reach of COPe-support, as well as a need for greater awareness among professionals who have contact with carers. In line with previous research [41], the need for more proactive approaches from professionals and services to identify and refer carers were highlighted, such as potential screening for the well-being of carers and signposting to COPe-support [32].

In any future rollout of COPe-support, it is imperative to consider the revision and refinement of the content as much as the facilitation of the minimally guided web-based intervention holistically to keep the participants engaged, to induce the anticipated impact [7]. Further scaling-up implementation of COPe-support and similar interventions also needs to carefully consider what constitutes the optimal group size and setup for a multicomponent web-based object, including closed forums catering for numerous users with varying use and participation profiles and a more flexible time frame to suit the ongoing needs of carers. Some participants also highlighted a desire for blended services—that is, COPe-support being adjunctive to, rather than a replacement of, in-person support. Indeed, a blended approach could foster the discussed benefits of both web-based and face-to-face support, as well as provide carers with options to cater for their needs and preferences.

### Strengths and Limitations

We considered the sample of 35 carers interviewed for this study as a strength, as this contributed to a wide variation in user experience and use from carers with different relationships with individuals with psychosis and in different caregiving situations. Having multiple researchers to independently code and analyze the rich data led to unanimous results and increased the rigor and reflexivity of the study [36,42]. The study results allow us to understand how carers engaged with COPe-support, what helped or hindered their engagement, and how using it affected themselves. Through these results, we underscored that carers’ experiences of COPe-support were shaped by a range of demographic and web-based health literacy factors, in addition to the intervention design and delivery itself. To ensure that users obtain the intended benefits of the COPe-support in the future rollout, it is imperative to consider how best to engage a wide variety of users to use all its essential ingredients [7,14].

This study had several limitations. Although we aimed to interview carers after completion of outcome data collection at 8 months, some carers had stopped using COPe-support earlier than the study duration and hence found it difficult to recall their experience with the intervention in detail. Although we strived to invite participants with low use and those from ethnic minority backgrounds for the interviews, such populations remained underrepresented (in the overall trial and this study) [43,44]. Our interviewees may have been positively biased in their views surrounding the intervention and study. It could be valuable to extend future work to explore reasons for nonenrollment among potential participants within services where the intervention was advertised, yet they chose not to take part.

### Conclusions

Overall, this qualitative interview study captured the experiences of carers of using the web-based intervention COPe-support. The variation in responses among active and passive users captured the carer’s perception of COPe-support. Notably, support and engagement with peers and experts were appreciated for meeting and validating the needs of carers, and the importance of usability ease, personalization, convenience, and safety were discussed. Further work is required to develop COPe-support based on these suggestions and explore the steps for optimal implementation.

## Appendix

Multimedia Appendix 1 Interview topic guide.

Multimedia Appendix 2 Summary of themes and subthemes.

## Abbreviations

COPe-support: Carers fOr People with Psychosis e-support
RCT: randomized controlled trial
