# Supplementary material for: Perceived Acceptability and Experiences of a Digital Psychoeducation and Peer Support Intervention (COPe-support): Interview Study With Carers Supporting Individuals With Psychosis
Source: J Med Internet Res. 2022 Feb 2;24(2):e27781. doi: 10.2196/27781 (PMC8851336; doi:10.2196/27781)
Supplement: Multimedia Appendix 1 [file jmir_v24i2e27781_app1.docx]

RCT of COPe-support online resource for carers

- Post intervention individual interview on experiences of using COPe-support

# Welcome & explanation

“Thank you for giving up some of your time to talk to me today. The length of the interview is entirely up to you but it will probably last approximately 30 minutes. I’ll ask you about your experiences of using the resource, any particular strengths and weakness of the resource, and lastly your views on ways to improve it for future/ other users.”

# Guidelines

“There are no right or wrong answers, what I am interested in is your own experiences and views. I will not ask you about details of the individual you support; rather the focus of this interview is upon you and your experiences.”

# Obtaining consent

“Have you read through the information sheet about the study?”

“Do you have any questions before we begin?” [Interviewer answers any questions]

“Can I ask you to read and sign the consent form to say that you agree to take part in the study and for it to be audio-recorded in order to ensure I have an accurate record of our discussion. All identifying information will be removed during transcription and once the interview has been fully transcribed and analysed the recording will be wiped clean.” [Interviewee reads & signs consent form]

**_________________________________________________________________________**

**Semi-structured questions/prompts**

1. Please tell me how you found the online resource?
2. Which parts/ elements you found most helpful for you?
3. Which parts/ elements were less / least helpful for you?
4. Was there any particular features/ strategies that engaged you/ kept you using it?

(prompts include: weekly reminders? Is smart-phone friendly important for you? Ground rules? Moderaton?)

1. On the contrary, did you encounter any problems that discouraged you from using it as much as you liked?
2. How often/frequent you used the resource? and roughly how much time over the 4 months?

(Did you think 4 months’ of using it allow you adequate time to get through all the content? Practice skills and integrate new skills into life)

1. Is there any change in you since using the resource? It could be anything from how your feel, what you think about your own mental health? Relationships or communication with your loved one?
2. Have you got any suggestions on ways to improve the resource?
3. Is there any other points you’d like to raise about your experiences and views on the resource?
