# Supplementary material for: Perceived Acceptability and Experiences of a Digital Psychoeducation and Peer Support Intervention (COPe-support): Interview Study With Carers Supporting Individuals With Psychosis
Source: J Med Internet Res. 2022 Feb 2;24(2):e27781. doi: 10.2196/27781 (PMC8851336; doi:10.2196/27781)
Supplement: Multimedia Appendix 2 [file jmir_v24i2e27781_app2.docx]

**Supplementary 2. Brief summary of the identified themes and sub-themes**

| Themes and sub-themes | Brief Summary |
| --- | --- |
| Remote flexible and personalized | Covers the experiences and perspectives of carers using COPe-support, with particular regards to navigation, safety, and usability. |
| Personalization | Carers valued being able to pick the content they viewed and their own pseudonym. Some participants wanted more choice regarding forum communication. |
| Convenience and flexibility | Carers appreciated the convenience of 24/7 access to information and experts. Carers also appreciated the flexibility of the intervention including having autonomy over usage/posting. |
| Moderation, safety and anonymity | Overall, the carers shared a sense of feeling safe on COPe-support. Anonymity helped carers to feel more comfortable interacting on the platform and protect the privacy of loved ones. Carers appreciated the ground rules for the forum and the forum moderation to help to keep it a safe place – some expressed a preference for knowledgeable, professional moderation over hypothetical carer moderation to dispel potentially misguided beliefs and manage disagreements. The weekly emails also had a positive impact. |
| Usability | Mixed experiences of usability were shared. Some felt confident given their computer literacy and previous experience, whilst others described barriers such as age and poor computer literacy. Being able to use COPe-support on difference devices was appreciated and most carers described an adjustment period whereby their confidence in navigating the intervention grew with time. |
| Recognition of different user types | Many carers were aware of different user types on COPe-support, namely active and passive users. Some active users felt the peer/expert forums made the intervention powerful. Most passive users were aware they had not posted and found reading the forum posts from other carers useful and knowing the forums were there if needed comforting in itself. |
| Impacts on well-being and outlook on caregiving | Represents the impacts on well-being and outlook on caregiving experienced from participating in COPe-support. |
| Knowledge, self-confidence and empowerment | Many carers felt COPe-support provided comprehensive, relevant and helpful information across several topics, which was useful for both new carers and supplemented the resources of carers with existing knowledge. However, some carers found the information generic, outdated, repetitive or initially overwhelming. Most carers felt the experts communicated in an appropriate and understandable way, yet some felt responses were vague as the experts did not know their full situations. That said, the information provided had empowered carers to seek further conversations with professionals caring for their loved one and increased their self-confidence regarding their caregiving abilities. |
| Supportive peer community | Carers expressed a sense of belonging and solidarity through the supportive peer community, which fostered mutual understanding and reduced isolation. Carers appreciated the opportunity to learn from and help other carers. Moreover, the peer community helped to normalise concerns and stigmatised psychosis-related topics, as well as helped carers to feel reassured, hopeful, and less overwhelmed. |
| Improved well-being | Carers recognised that COPe-support was specifically designed for them, and showed the importance of self-care and healthier routines. The intervention fostered feelings of acknowledgement and validation of their own support needs, as well as facilitated personal space and time to reflect on their personal journey as carers. |
| Future implementation and integration with existing services | Reflects carers’ perspectives surrounding COPe-support’s future implementation, integration with existing services and suggested improvements. |
| Comparison with face-to-face support | Comparisons with face-to-face support for carers were mixed. Barriers to face-to-face support (geographical factors, family life, funding, and time constraints) and online interventions (age and the desire to personally meet carers) were discussed. Some expressed a preference, whilst others felt a blended approach would be ideal. |
| Integration with other services | Given funding restrictions on existing services, carers highlighted the benefits of implementing COPe-support. Some carers found their positive experiences of using COPe-support had motivated them to utilise other services for carers. Some felt COPe-support was solely sufficient for their needs, whilst others emphasised it should be used as an adjunctive to existing services rather than a replacement. |
| Continuous access | Views on access time were mixed. Some felt they had received access to COPe-support for the right amount of time to gain optimal benefits. However, some participants desired longer usage time to be able to access the information and support in future times of need. To enable this, some participants suggested having continual access to COPe-support or being able to self-refer back in. |
| Greater advertisement and reach | Some carers felt COPe-support needed greater advertisement. Recommendations for increasing reach included utilising more advertising and promotion routes and increasing awareness of COPe-support amongst health and social care professionals. |
| User suggested improvements | Suggested improvements included: fewer chunks of text, more graphics/visual aids, ‘see more’ drop down options, content warnings, a chat room or befriender element, brief carer profiles, frequently/recently viewed buttons on the forums, additional prompts following periods of inactivity, rolling discussion topics, and alternative engagement options such as emoji reactions. |
